# Supplementary figures and images for: Population Genetic Structure of Citrus Tatter Leaf Virus in Zhejiang Province, China
Source: Viruses. 2025 Jun 27;17(7):909. doi: 10.3390/v17070909 (PMC12299479; doi:10.3390/v17070909)

Fig. S1

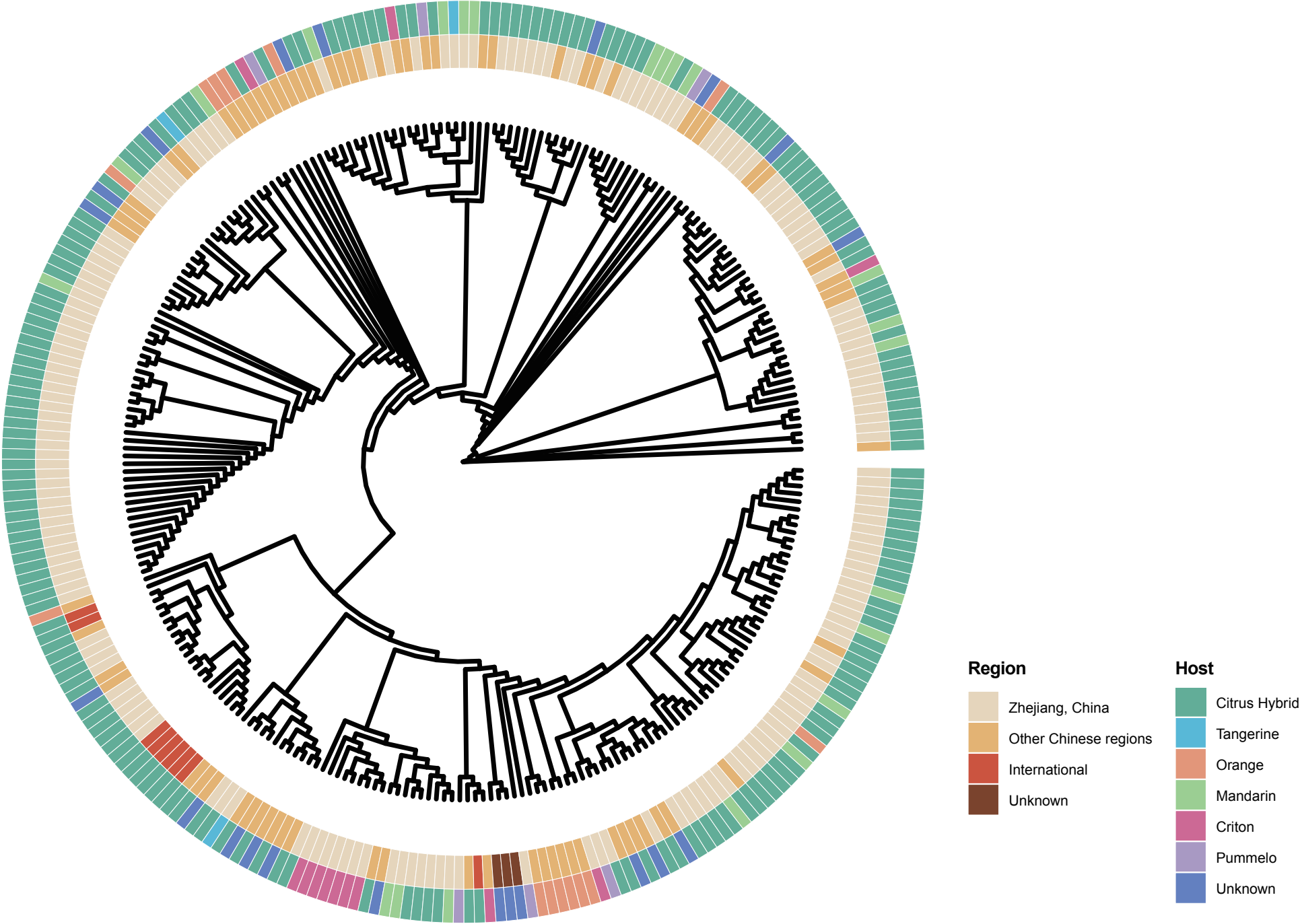

Supplement: Supplementary file 1 [file viruses-17-00909-s001.zip › Figure S1. R1_updated.pdf]

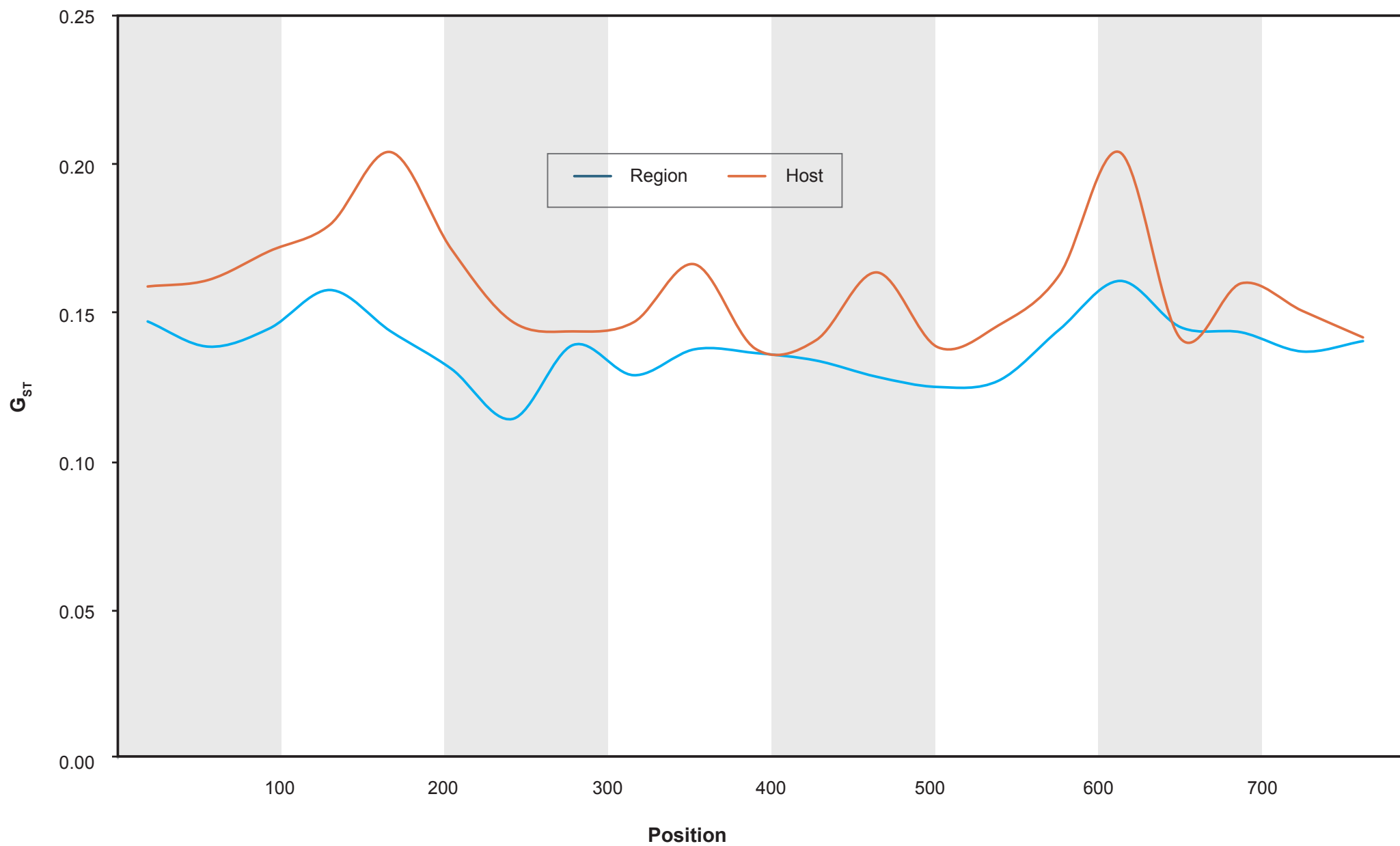

Supplement: Supplementary file 1 [file viruses-17-00909-s001.zip › Figure S2.pdf]

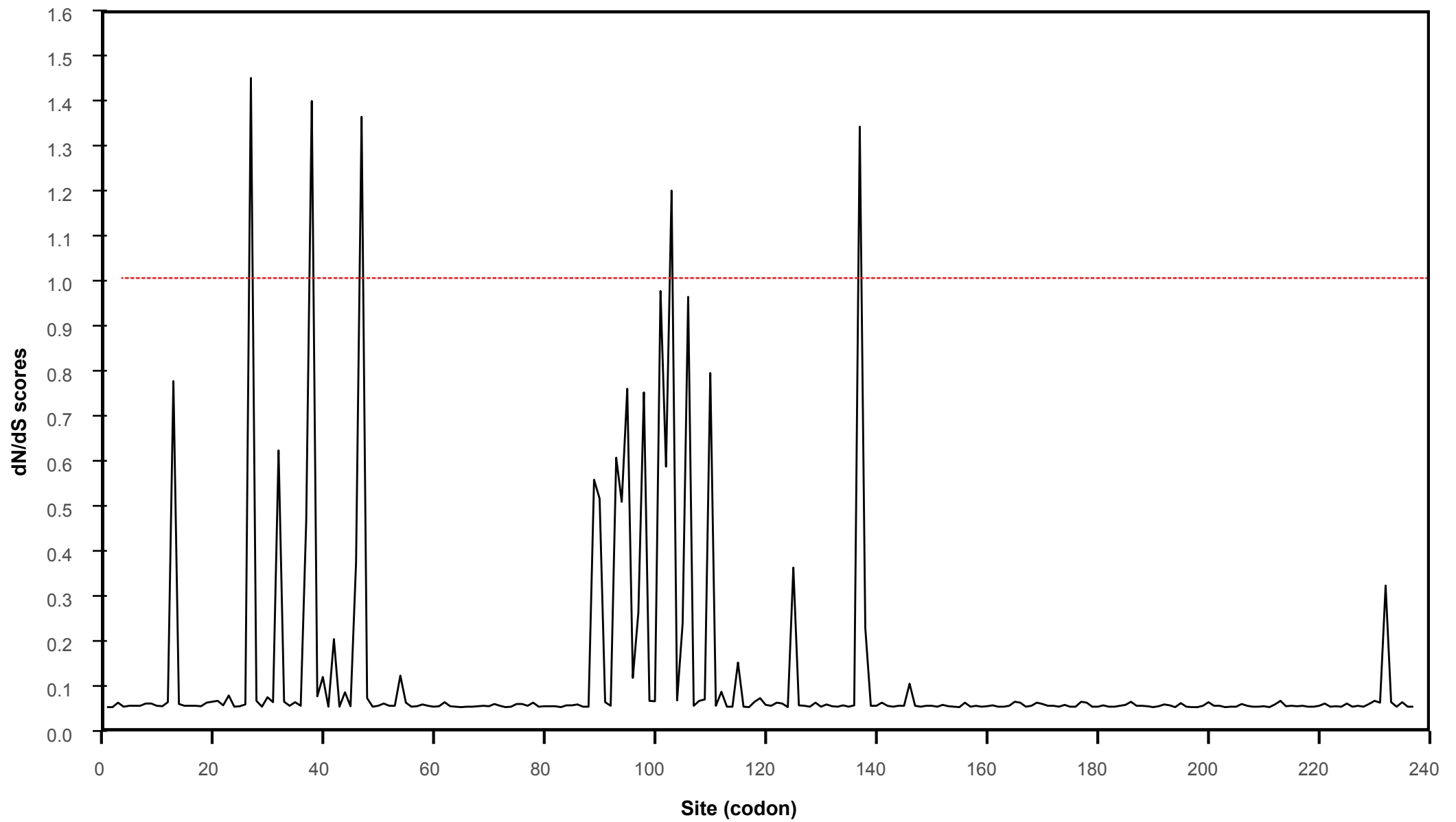

Supplement: Supplementary file 1 [file viruses-17-00909-s001.zip › Figure S3.pdf]

a. Zhejiang isolate

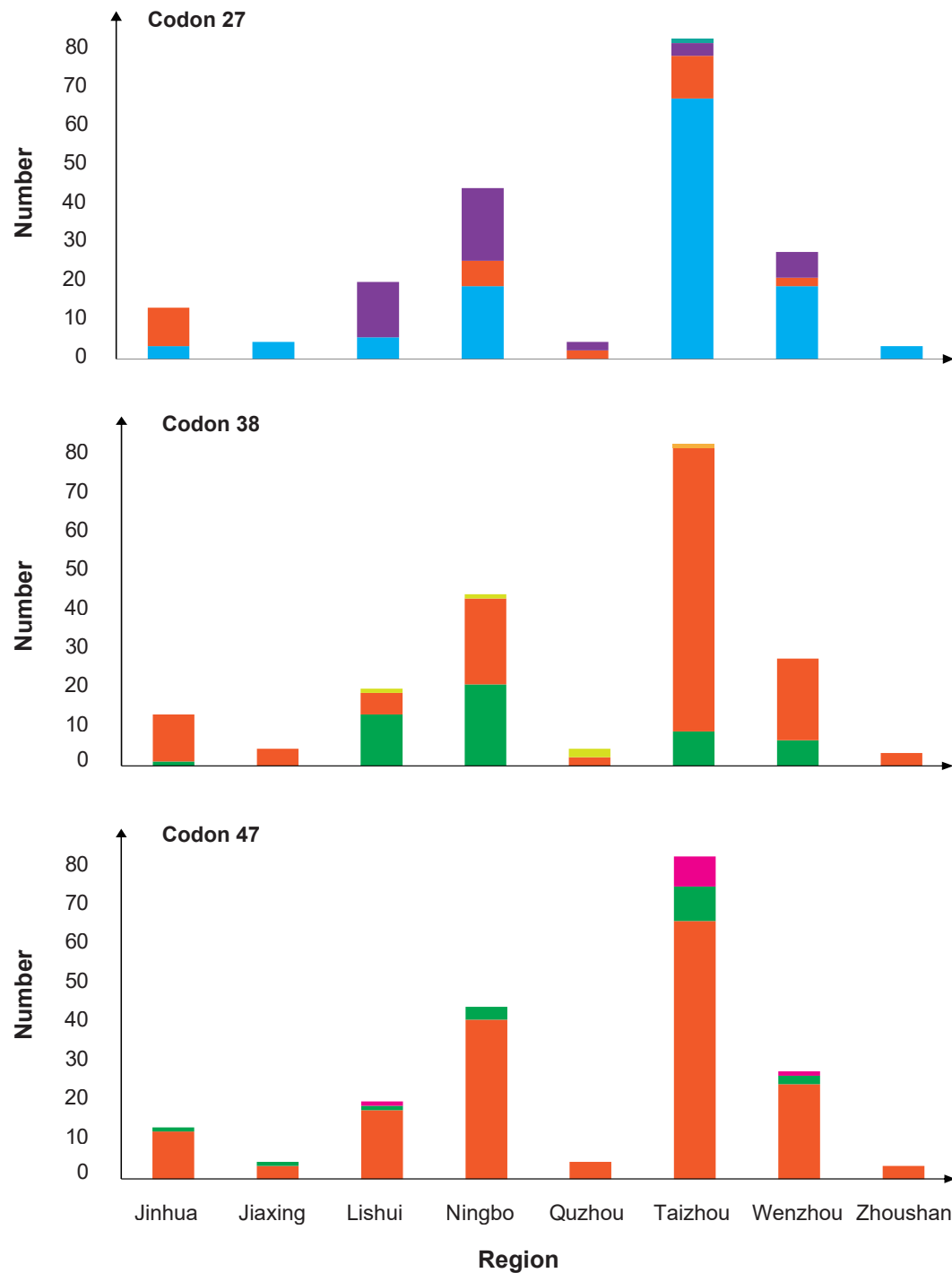

b. Global isolate

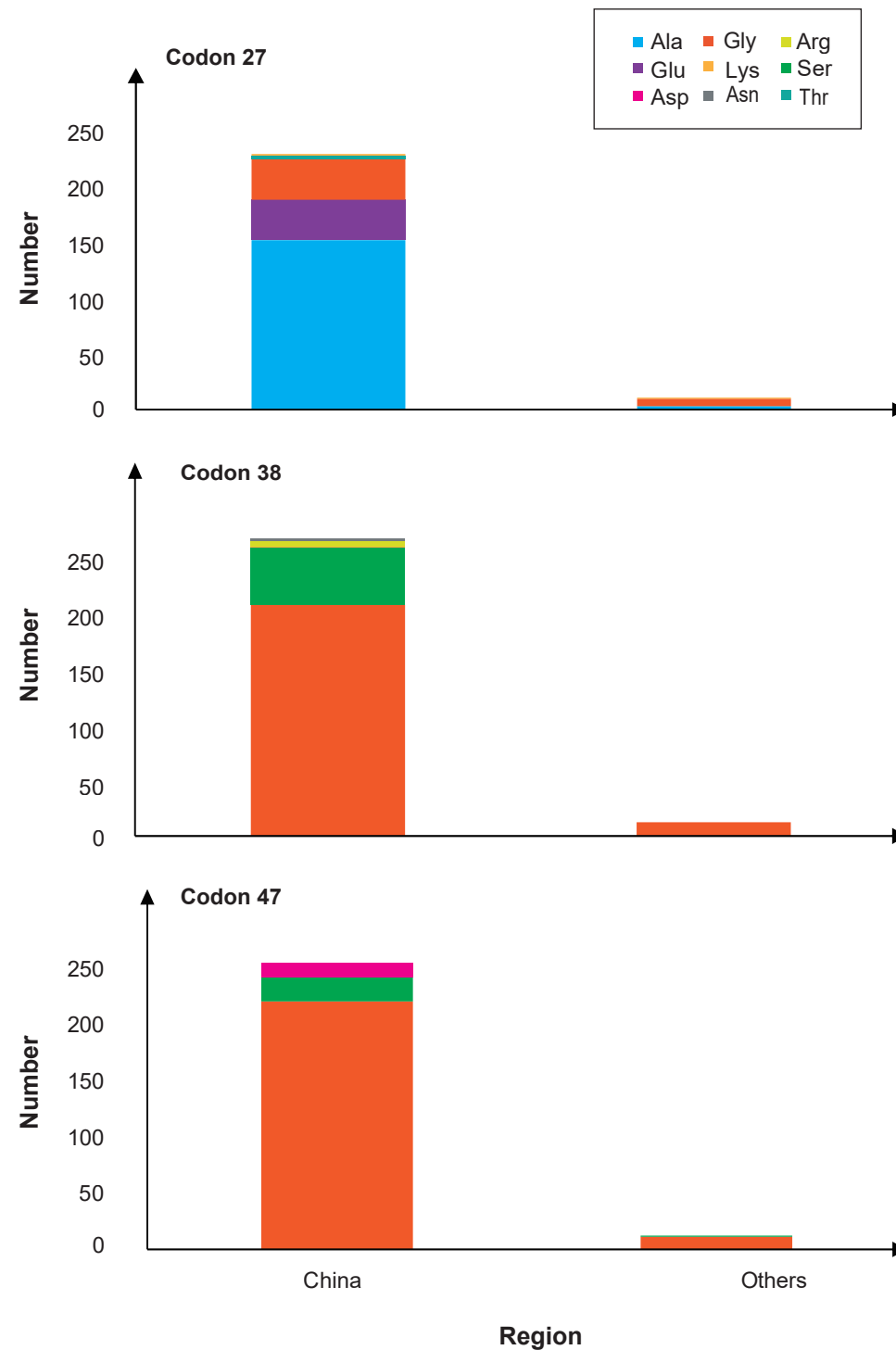

Supplement: Supplementary file 1 [file viruses-17-00909-s001.zip › Figure S4. R1.pdf]
